# Factor VII, EPCR, aPC Modulators: novel treatment for neuroinflammation

**DOI:** 10.1186/s12974-022-02505-y

**Published:** 2022-06-11

**Authors:** Valery Golderman, Marina Ben-Shimon, Nicola Maggio, Amir Dori, Shany Guly Gofrit, Shani Berkowitz, Lamis Qassim, Avital Artan-Furman, Talya Zeimer, Joab Chapman, Efrat Shavit-Stein

**Affiliations:** 1 Department of Neurology, The Chaim Sheba Medical Center, 52621 Ramat Gan, Israel; 2 Neurology and Neurosurgery, Sackler Faculty of Medicine, Tel Aviv University, Tel Aviv, Israel; 3 Sackler Faculty of Medicine, Joseph Sagol Neuroscience Center, Tel Aviv University, Tel Aviv, Israel; 4 Talpiot Medical Leadership Program, The Chaim Sheba Medical Center, Ramat Gan, Israel; 5 Sackler Faculty of Medicine, Robert and Martha Harden Chair in Mental and Neurological Diseases, Tel Aviv University, Tel Aviv, Israel; 6 The TELEM Rubin Excellence in Biomedical Research Program, The Chaim Sheba Medical Center, Ramat Gan, Israel

**Keywords:** Neuroinflammation, Coagulation, LPS, mTBI, Microglia, Thrombin, aPC

## Abstract

**Background:**

Inflammation and coagulation are linked and pathogenic in neuroinflammatory diseases. Protease-activated receptor 1 (PAR1) can be activated both by thrombin, inducing increased inflammation, and activated protein C (aPC), inducing decreased inflammation. Modulation of the aPC-PAR1 pathway may prevent the neuroinflammation associated with PAR1 over-activation.

**Methods:**

We synthesized a group of novel molecules based on the binding site of FVII/aPC to the endothelial protein C receptor (EPCR). These molecules modulate the FVII/aPC-EPCR pathway and are therefore named FEAMs—Factor VII, EPCR, aPC Modulators. We studied the molecular and behavioral effects of a selected FEAM in neuroinflammation models in-vitro and in-vivo.

**Results:**

In a lipopolysaccharide (LPS) induced in-vitro model, neuroinflammation leads to increased thrombin activity compared to control (2.7 ± 0.11 and 2.23 ± 0.13 mU/ml, respectively, *p* = 0.01) and decreased aPC activity (0.57 ± 0.01 and 1.00 ± 0.02, respectively, *p* < 0.0001). In addition, increased phosphorylated extracellular regulated kinase (pERK) (0.99 ± 0.13, 1.39 ± 0.14, control and LPS, *p* < 0.04) and protein kinase B (pAKT) (1.00 ± 0.09, 2.83 ± 0.81, control and LPS, *p* < 0.0002) levels indicate PAR1 overactivation, which leads to increased tumor necrosis factor-alpha (TNF-α) level (1.00 ± 0.04, 1.35 ± 0.12, control and LPS, *p* = 0.02). In a minimal traumatic brain injury (mTBI) induced neuroinflammation in-vivo model in mice, increased thrombin activity, PAR1 activation, and TNF-α levels were measured. Additionally, significant memory impairment, as indicated by a lower recognition index in the Novel Object Recognition (NOR) test and Y-maze test (NOR: 0.19 ± 0.06, -0.07 ± 0.09, *p* = 0.03. Y-Maze: 0.50 ± 0.03, 0.23 ± 0.09, *p* = 0.02 control and mTBI, respectively), as well as hypersensitivity by hot-plate latency (16.6 ± 0.89, 12.8 ± 0.56 s, control and mTBI, *p* = 0.01), were seen. FEAM prevented most of the molecular and behavioral negative effects of neuroinflammation in-vitro and in-vivo, most likely through EPCR-PAR1 interactions.

**Conclusion:**

FEAM is a promising tool to study neuroinflammation and a potential treatment for a variety of neuroinflammatory diseases.

## Introduction

Numerous studies describe the interactions between the inflammation and the coagulation systems, with enhanced inflammation leading to thrombosis and vice versa [1, 2]. This interaction is especially pronounced in the coagulopathy seen in severe sepsis [3] and infections leading to cytokine storms such as in COVID-19 [4]. Neuroinflammation is involved in many pathologies [5–7], as well as in diseases with coagulation dysfunction [8]. In addition to the role of coagulation in neuroinflammation, coagulation system proteins are also directly involved in neuronal function and survival [9–11].

Protease-activated receptor 1 (PAR1) is a pivotal G-protein coupled receptor which can be activated by several proteases, including thrombin, activated protein C (aPC), and FVIIa [12]. PAR1 activation by thrombin promotes human platelet aggregation during the process of clot formation [13]. In neuronal tissue, PAR1 functions as a molecular switch, with different effects induced by different activating proteases. Activation of PAR1 by high levels of thrombin was observed following neuro-pathologies such as stroke [14], minimal traumatic brain injury (mTBI) [15], and diabetic neuropathy [16]. Inhibition of PAR1 activation by high thrombin levels was found to be protective [16–18]. However, PAR1 activation by low thrombin levels can activate a neuroprotective phenotype in astrocytes [19], suggesting that PAR1 activation may have contrasting effects.

Activation of PAR1 by the aPC involves PC activation by its receptor, the endothelial protein C receptor (EPCR). The aPC-EPCR complex then cleaves and activates PAR1 [20]. PAR1 activation by the aPC-EPCR complex promotes neurogenesis in the ischemic brain [21], inhibits activation and translocation of nuclear factor-κB, and reduces blood–brain barrier permeability [22]. aPC neuroprotective properties are a target for pharmacological intervention.

Uncontrolled coagulation in sepsis, accompanied by aPC consumption, led to the use of recombinant aPC as a treatment. However, previous attempts to treat sepsis with aPC failed in post-marketing evaluation (the PROWESS-SHOCK trial) [23], and the drug was taken off the market.

The positive effects of aPC on ischemic neuronal tissue led to the development of the 3K3A-APC by the point replacement of Lys by Ala, abolishing the anti-coagulation effects but preserving PAR1 activation. The 3K3A-APC provided promising results in ischemia models in mice and human stroke patients [24].

Modulation of PAR1 activity is therefore a powerful pharmacologic target in neuro-pathologies. Therefore, we synthesized a set of molecules, based on the FVII/PC-EPCR interaction sequence and named them FEAMs (Factor VIIa, EPCR, aPC Modulators). FEAMs are designed with a backbone of 4 to 7 amino-acids gamma carboxylated modified (important GLA domain). In the present study, we examined four FEAM molecules (1–4) and selected the optimal molecule based on its effect on microglial cell proliferation and aPC activity. The chosen molecule was first tested for safety and then in in-vitro and in-vivo neuroinflammation models*.* FEAM improved LPS induced molecular changes in an in-vitro N9 cell model and mTBI induced molecular and behavioral changes in an in-vivo mice model.

## Methods

### Molecule design

FEAM1-4 were designed in our lab and prepared by Sigma Custome Synthesis Unit Israel (> 98% purity) based on our custom special order. The amino acids sequences were selected according to previous publications [25] that reported the important and common regions for aPC and FVII interaction with EPCR. The N-terminus of FEAMs consists of the TOSYL (TOS) group which protects from endopeptidase cleavage while the C-terminus consists of amide modification for molecule stability (Table 2).

### Cell culture

N9 mouse microglial cells were grown in Dulbecco’s modified Eagle’s medium (DMEM; Bet Haemek, Biological Industries, Israel) supplemented with 10% fetal bovine serum (Bet Haemek, Biological Industries, Israel), 1% l-Glutamine (Bet Haemek, Biological Industries, Israel) and 0.1% penicillin and streptomycin (Bet Haemek, Biological Industries, Israel). The cells were grown in a 37 °C and 5% CO_2_-humidified atmosphere.

### Cell proliferation

Cell proliferation was evaluated using a 2.3-Bis-(2-methoxy-4-nitro-5-sulfophenyl)-2H-tetrazolium-5-carboxyanilide salt (XTT)-based cell proliferation assay kit (Bet Haemek, Biological Industries, Israel). N9 cells were seeded (1×10^5^cells/ml, 200 µl/well) and allowed to attach for 24 h. The medium was then replaced by serum-free DMEM (100 µl) containing lipopolysaccharide (LPS, a component of the bacterial wall 0.1 µg/ml, O111:B4, Sigma, L4130) and/or FEAMs (10 µM). Following 24 h, XTT solution (50 μl) was added to each well and incubated for 3 h at 37 °C. The optical density (OD) of each well was recorded at 450 nm and 630 nm in a microplate reader (Infinite M200, Tecan, Durham, NC, USA).

### aPC activity

aPC activity was assessed using a fluorogenic substrate as described previously [26]. Purified aPC activity was measured using a reference standard PC (88%, Technoclone, 5341013) that was activated by Protac (Technoclone, 5346212) for 5 min and placed in a black 96-well microplate with and without FEAM (10 µM). Substrate (Pyr-Pro-Arg-AMC, 20 µM, GL Biochem Shanghai Ltd.) was added immediately and the fluorescence was measured using a microplate reader (Infinite M200, Tecan, Durham, NC, USA) with excitation and emission filters of 360 ± 35 and 460 ± 35 nm, respectively. The cleavage of the substrate was measured at 37 °C, every 2 min for 25 cycles. For in-vitro experiments, N9 cells were seeded (1×10^5^cells/ml, 200 µl/well) and allowed to attach for 24 h. The medium was then replaced by serum-free DMEM (100 µl) with LPS (0.1 µg/ml) and/or FEAMs (10 µM). 24 h later, the medium was transferred to a black 96-well microplate. Substrate (20 µM) with inhibitors (alpha-naphthylsulphonylglycyl-4-amidinophenylalanine piperidine (NAPAP)—1 µM, Santa Cruz, sc-208083, Apixaban—1 µM, Selleckchem, S1593) was added immediately and the fluorescence was measured. For in-vivo experiments, mice were sacrificed using lethal phenobarbital injection (CTS Chemical Industries ltd, pentobarbital sodium 200 mg/ml, 300 mg/kg) followed by rapid decapitation. The right hippocampus was removed immediately after dissection and placed in a 96 well black plate containing buffer (in mM: 50 TRIS/HCl pH 8.0, 150 NaCl, 1 CaCl_2,_ and 0.1% BSA). Substrate (20 µM) with inhibitors (bestatin, 0.1 mg/ml, Cayman Chemical Company; USA, prolyl endopeptidase inhibitor, 0.2 mM, Merck; Germany, NAPAP—1 µM, Apixaban—1 µM) was added immediately and the fluorescence was measured. The results are presented as a linear increase of fluorescence intensity over time, relative to control.

### FXa activity

FXa activity was measured using a fluorometric assay based on the cleavage rate of the synthetic substrate Boc-Ile-Glu-Gly-Arg-AMC (50 μM, I-1100; Bachem, Bubendorf, Switzerland). Commercial bovine FXa (Haematologic Technologies, BCXA-1060) was diluted in buffer (in mM: 50 TRIS/HCl pH 8.0, 150 NaCl, 1 CaCl2, and 0.1% BSA) and placed in a 96 well black plate with and without FEAM (10 µM). The substrate (50 μM) was added to each well (the final volume in each well was 100 μl). The cleavage of the substrate was measured using a microplate reader at 37 °C, every 2 min for 25 cycles. The results are presented as a linear increase of fluorescence intensity over time, relative to control.

### Thrombin activity

Thrombin activity was measured using a fluorometric assay based on the cleavage rate of the synthetic substrate Boc-Asp (OBzl)-ProArg-AMC (I-1560; Bachem, Bubendorf, Switzerland) as described previously [27]. Purified thrombin activity was measured using bovine thrombin (0.05U, Sigma, T4648) that was diluted in buffer (in mM: 50 TRIS/HCl pH 8.0, 150 NaCl, 1 CaCl2, and 0.1% BSA) and placed in a black 96-well microplate with and without FEAM (10 µM). Substrate (14 μM) was added immediately, and the fluorescence was measured using a microplate reader. For in-vitro experiments, N9 cells were seeded (1X10^5^cells/ml, 200 µl/well) and allowed to attach for 24 h. The medium was then replaced by serum-free DMEM (100 µl) with LPS (0.1 µg/ml) and/or FEAMs (10 µM). 24 h later, the medium was transferred to a black 96-well microplate. The substrate (14 μM), containing aminopeptidase and prolyl endopeptidase inhibitors (bestatin, 0.1 mg/ml and prolyl endopeptidase inhibitor, 0.2 mM), was added to each well. The final volume in each well was 100 μl. For in-vivo experiments, mice were sacrificed using lethal phenobarbital injection followed by rapid decapitation. The right dorsal hippocampus was removed immediately after dissection and placed in a 96 well black plate containing buffer (in mM: 50 TRIS/HCl pH 8.0, 150 NaCl, 1 CaCl_2_, and 0.1% BSA). The substrate (14 μM), containing aminopeptidase and prolyl endopeptidase inhibitors (bestatin, 0.1 mg/ml, and prolyl endopeptidase inhibitor, 0.2 mM), was added to each well. The final volume in each well was 100 μl, not including the brain slice. Bovine thrombin was used for calibration.

### Coagulation in plasma

HemosIL normal control (Instrumentation Laboratory) was used as human normal pooled citrated plasma from healthy donors. The lyophilized human plasma containing buffer, stabilizers, and preservatives, was reconstituted using standard laboratory methods. The plasma was exposed in-vitro to indicated concentrations of FEAM for 10 min. Next, prothrombin time (PT), activated partial thromboplastin time (aPTT), thrombin time (TT), anti-factor Xa (anti-Xa) assay (measures the residual FXa activity/ FXa inhibition), and aPC resistance assay (which measures the potential direct effect on aPC enzymatic activity) were measured. All tests were carried out with an ACLTOP® 500 autoanalyzer at the Sheba Medical Center (Israel) MegaLab according to standard operating procedures (SOP). The results represent an average of 5 measures.

### Animals

Adult ICR male mice (Envigo, Jerusalem, Israel), 8–10 weeks old, were housed in standard conditions and fed standard diet and water available ad libitum. The ambient temperature was set to 22 to 23 °C with day/night light control. The study protocols were approved by the Sheba Medical Center Committee on the Use and Care of Animals (ANIM-1084-17) according to the ARRIVE Guidelines.

### Minimal traumatic brain injury (mTBI)

#### Experimental design

**Fig. 1:**
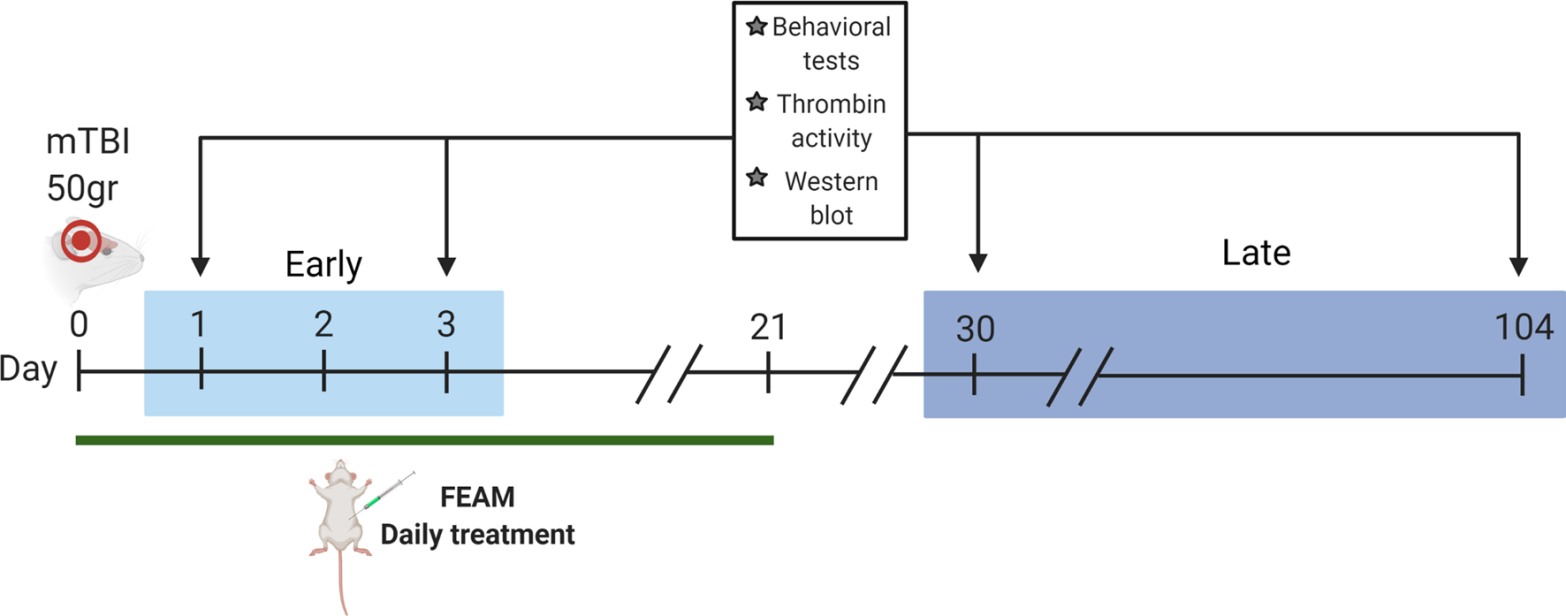
mTBI—experimental design. mTBI was induced at day 0 and the mice were assessed and sacrificed at four points in time: early points in time—1 and 3 days following mTBI, late points in time—30, and 104 days following mTBI. FEAM treatment was applied from day 0 to day 21. Created with BioRender.com

Mild traumatic brain injury was induced on day 0. Assessment and sacrifice of the animals were conducted at 4 points in time (1, 3, 30, 104 days, Fig. 1), 30 mice for each point (a total of 120 mice). At each point in time, 10 mice were allocated to each group:

(1) Control group—anesthetized only; (2) mTBI group—anesthetized + induced mTBI; (3) mTBI + FEAM group—anesthetized + induced mTBI + FEAM treatment.

FEAM treatment was applied intraperitoneally (I.P.) repeatedly at a concentration of 0.035 mg/kg. FEAM treatment was given daily for 21 days post mTBI induction. Mice were weighed frequently throughout the period of the experiment.

At each timepoint, behavior was assessed, and the mice were sacrificed using lethal I.P. phenobarbital injection (300 mg/kg) followed by rapid decapitation. The brain was removed, and the hippocampi were used for thrombin activity assay and western blot analysis.

#### mTBI induction

mTBI was carried out as previously described by Itzekson et al. [15]. mTBI was induced using a free weight drop concussive device. The device consisted of an 80-cm-high metal tube (13 mm in diameter) placed vertically over the head of the mouse. Minutes prior to the injury, the animals were slightly anesthetized by isoflurane (gaseous, 0.5 ml of isoflurane was applied to a cotton swab in a 3L desiccator, for up to 60 s, to form 4% isoflurane). Trauma was induced by a 50 g metal weight dropped down the metal tube on the right anterolateral side of the head (just anterior to the right ear). The mouse was placed on a sponge immobilization board which allowed head rotation following the impact, thus mimicking the natural condition of head rotation in a whiplash injury. Control mice were slightly anesthetized by isoflurane.

### Behavioral tests

Behavior was assessed at least one week following the arrival of the mice to the institutional animal facility to attenuate stress. The experiments were always conducted at the same time of day to avoid circadian cycle effects. All behavioral tasks were performed in polymethyl methacrylate apparatus. The arenas were cleaned and dried between each session. The tasks were performed in a room dedicated to long-term behavioral procedures and conducted by the same experimenter who was blinded to the animal group allocation. During the behavioral experiments, all efforts were made to minimize interruptions such as door openings and the sights and smells of people. During the same behavioral tasks, the apparatus surrounding area remained unchanged to avoid un-balanced cues in spatial memory tasks. Experimental sessions were recorded via a fixed camera placed above the apparatus and the real-time acquisition of mice positions in the arena was recorded by EthoVision XT 11.5v (Noldus) video tracking program.

#### Novel object recognition (NOR)

A novel object recognition test was used to assess recognition memory, as previously described [28]. Initially, mice were individually habituated to an open field box (47 × 47 × 29 cm) for 5 min, 24 h before testing. During the acquisition phase, two identical objects, which were sufficiently heavy and high to ensure that mice could neither move nor climb over them, were placed in a symmetric position within the chamber. The test was conducted 24 h following the acquisition. During the test, one of the objects in the arena was substituted by a novel one, and exploratory behavior was again assessed for 5 min (discrimination phase). All objects were thoroughly cleansed (70% ethanol) between sessions to preclude odor recognition. The objects applied in all trials were common objects that differ in shape and texture and stand out against the background: a Lego brick tower (8-cm high and 3.2-cm wide, built-in white, blue, yellow, red, and green bricks) and a Falcon tissue culture flask filled with sand (9.5 cm high, 2.5 cm deep and 5.5 cm wide, transparent plastic with a blue bottle cap), as was previously described [29]. Exploration was defined as directing the nose toward the object at a distance less than or equal to 4 cm. Successful recognition was revealed by preferential exploration of the novel object. Discrimination of visual novelty was assessed by a discrimination index defined as (the exploration time devoted to the novel object-the time devoted to the familiar object)/(the total amount of exploration of the novel + familiar objects).

#### Y-maze

The Y-maze was used to assess spatial memory [30]. The Y-shaped apertures consisted of three arms. Spatial cues included vertical tape positioned at the entrance to the familiar arm and a triangle to the novel arm. Mice were placed in one arm of the Y-maze (entrance arm) with one of the arms blocked off (novel arm). Mice were allowed to explore the start arm and remaining arm (familiar arm) for 5 min. After a 30 min break, mice were placed back in the entrance arm and allowed to explore all arms freely for 2 min. The time spent in each arm of the maze and the percentage of mice entering the novel arm first were recorded. Discrimination of novelty versus familiarity was assessed by calculating the discrimination index defined as (duration time in the novel arm—the duration time in the familiar arm)/(the total amount of exploration of the novel + familiar arms).

#### Hot-plate test

Hyper/hypoalgesia was evaluated using the hot plate test. Mice were placed in an acrylic glass cylinder on a heated stage, digitally maintained at 51 ± 0.1 °C. Time to heat response indicated by hind paw licking, shaking or jumping was measured. A maximum on-plate time was set to 30 s to prevent skin injury.

### Western blot analysis

Cells were seeded (1×10^6^ cells/flask) and allowed to attach for 24 h. The medium was then replaced by serum-free media with treatments LPS (0.1 µg/ml) with or without FEAM (10 µM). After 24 h the samples were collected on ice as follows: cells were washed with cold phosphate-buffered saline (PBS), radioimmunoprecipitation assay (RIPA) lysis buffer (in mM: 50 TRIS HCl pH 8.0, 150 NaCl, 1% NP-40, 0.5% Sodium Deoxycholate, 0.1% SDS) containing phosphatases and proteases inhibitors (protease inhibitors cocktail (1:100, 539134, Calbiochem), PMSF (2 mM, P7626, Sigma) and sodium orthovanadate (1 mM, S6508, Sigma)) was added and the cells were scraped and collected. The cells were incubated on ice for 10 min, centrifuged (16,000 *g*, 20 min, 4 °C) and the supernatant was collected for further analysis.

Hippocampal samples were homogenized in RIPA buffer and a protease inhibitor cocktail (1:100). The microcentrifuge tubes were placed in a bullet blender homogenizer (Next Advance) at maximum speed for 1 min. The homogenates were then centrifuged (13,000 *g*, 5 min, 4 °C). The supernatant was collected for further analysis.

Protein concentration was determined utilizing a bicinchoninic acid (BCA) assay. For each sample, 10–20 μg proteins were separated by SDS–polyacrylamide gel electrophoresis. The proteins were transferred onto nitrocellulose membranes. After transfer, the nitrocellulose membranes were rinsed briefly in TBST (in mM: 10 TRIS HCl pH 8.0, 150 NaCl, 0.01% Tween-20) and incubated in Ponceau S solution (0.5 [w/v] in 1% [v/v] acetic acid) for 2 min, followed by a brief rinse in TBST for optimal bands visibility. Ponceau bands were scanned and the membranes were washed with TBST until completely clean. Membranes were incubated overnight at 4 °C with antibodies as indicated in Table 1 and washed. Membranes were then incubated at room temperature with horseradish peroxidase-conjugated goat anti-rabbit/mouse antibody (1:10,000, Jackson Immunoresearch Laboratories). Protein bands were detected by a peroxidase-based ECL method. Upon detection, the membranes were stripped and re-incubated with mouse anti-actin antibody (1:10,000, MAB1501) or rabbit anti-AKT/ERK2 antibody and re-detected by ECL. The signal was developed using an enhanced chemiluminescent reagent (20-500-500, Biological Industries) and exposed to X-ray film (Fuji Photofilm, Bedfordshire). The quantification of protein band density on X-ray film was performed by Image J analysis PC software [31].

**Table 1:** Table of primary antibodies used in western blot method

Antibody	Dilution	Company (# catalog)
Rabbit anti-Protein C	1:400	Abbiotec (251142)
Rabbit anti Thrombin	1:400	Bioss (19142)
Rabbit anti-PAR1	1:500	Bioss (0828R)
Rabbit anti- TNFα	1:500	GeneTex (110520)
Mouse anti-pAKT	1:2000	Cell Signaling (2920)
Rabbit anti-AKT	1:2000	Cell Signaling (S473)
Mouse anti-pERK	1:10,000	Sigma (M8159)
Rabbit anti-ERK2	1:5000	R&D Dydtems (AF1230)

### Neurofilament (NfL)

The blood was collected immediately following decapitation. The collected blood was incubated in room temperature for 30 min and centrifuged (1500 g, 10 min, 25 °C). The serum was collected and stored at – 80 °C until the assay. Mouse serum NfL concentration was determined using the Simoa NfL assay as previously described [32] (Quanterix, Quanterix Corp, Boston, MA, USA) (UD2, UmanDiagnostics) in a Simoa instrument (Quanterix) using a bead-conjugated immunocomplex. The immunocomplex was applied to a multi-well array designed to enable imaging of every single bead. The average number of enzymes per bead (AEB) of samples was interpolated onto the calibrator curve constructed by AEB measurements on bovine NfL (UmanDiagnostics) serially diluted in assay diluent. Samples were analyzed using one batch of reagents and animal treatment information was blinded to the one performing the analysis.

### Statistics

Statistical analyses and graphs were conducted using GraphPad Prism (version 7.00 for Windows, GraphPad Software, La Jolla California USA, www.graphpad.com). Paired t-tests, one-way ANOVA, and two-way ANOVA followed by a post hoc test were applied on normally distributed data sets. One-way ANOVA was followed by either Dunnett’s or Tukey’s post hoc analyses. Two-way ANOVA was followed by Sidak’s post hoc analysis. Results of post analyses are presented only when ANOVA results show statistical significance. The Mann Whitney test was applied to non-normal distributed data sets. Results are expressed as mean ± SEM, *p* values < 0.05 were considered significant.

## Results

### *FEAM—molecule characterization and *in-vitro* inflammation model:*

**Fig. 2:**
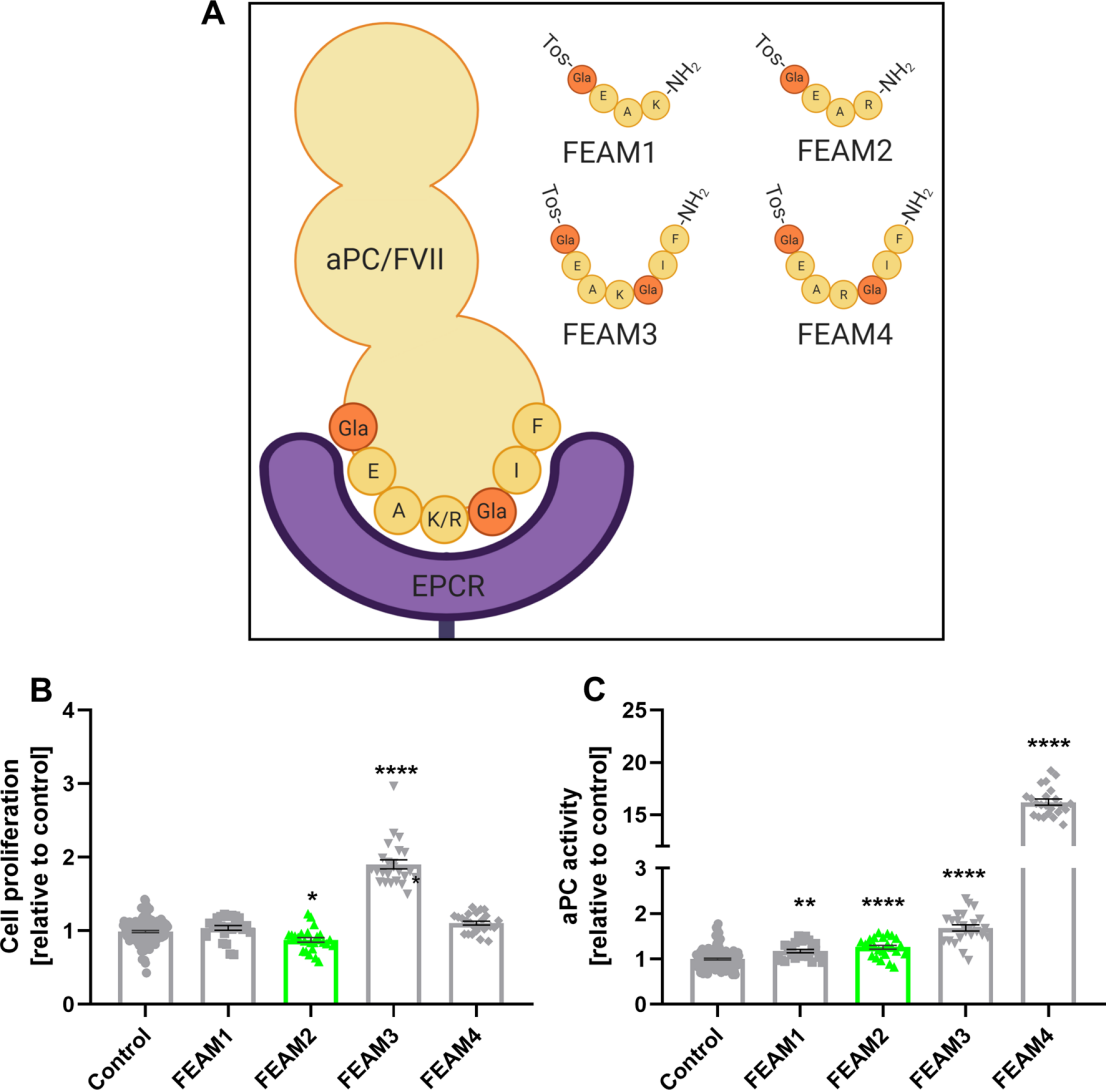
FEAMs molecules design rationale and their in-vitro effects on proliferation and aPC activity in N9 microglial cells: **A** FEAM1-4 structure: the structure of FEAM 1–4 is based on the binding sequence of human aPC/FVII to EPCR. Tosyl group on the N-terminal protects from endopeptidases, and amide modification on the C-terminal contributes to stability. Created with BioRender.com. **B** Cell proliferation: N9 cells proliferation in the presence of FEAM1-4. In the presence of FEAM2 (green), N9 cells showed significantly lower cell proliferation while, in the presence of FEAM3, N9 cells showed significantly higher cell proliferation, compared to control. *N* = 24–120. **C** aPC activity: N9 cells aPC activity was examined in the presence of FEAM1-4. N9 cells showed significantly higher aPC activity in the presence of FEAM1-4, compared to control. *N* = 23–113. Results are presented as mean ± SEM, *p < 0.05, **p < 0.01, ****p < 0.0001

We designed four molecules, based on the human aPC/EPCR and FVII/EPCR binding domain sequence (Table 2, Fig. 2A). We applied the molecules to N9 cells for 24 h and examined their effect on cell proliferation and secreted aPC activity. We found that FEAM2 significantly decreased cell proliferation compared to control (0.87 ± 0.03, 1.00 ± 0.01, respectively, *p* = 0.01, Fig. 2B), and FEAM3 significantly increased cell proliferation, compared to control (1.9 ± 0.06, *p* < 0.0001, Fig. 2B). In addition, all the molecules significantly increased aPC activity compared to control (*p* < 0.0001), and FEAM4 specifically showed the highest increase (16.2 ± 0.28, 1.00 ± 0.02, respectively, *p* < 0.0001, Fig. 2C). Based on these experiments, we decided to further study FEAM2 (which will be designated as FEAM), the only molecule that decreased cell proliferation and increased secreted aPC activity, as a treatment for neuroinflammation.

**Table 2:** The sequence of FEAM 1–4 and the corresponding region in human aPC/FVII

Molecules	Formula	Region
FEAM1	Tos-{gla}-EAK-NH2	haPC 25–28
FEAM2	Tos-{gla}-EAR-NH2	hFVII 25–28
FEAM3	Tos-{gla}-EAK-{gla}-IF-NH2	haPC 25–31
FEAM4	Tos-{gla}-EAR-{gla}-IF-NH2	hFVII 25–31

**Fig. 3:**
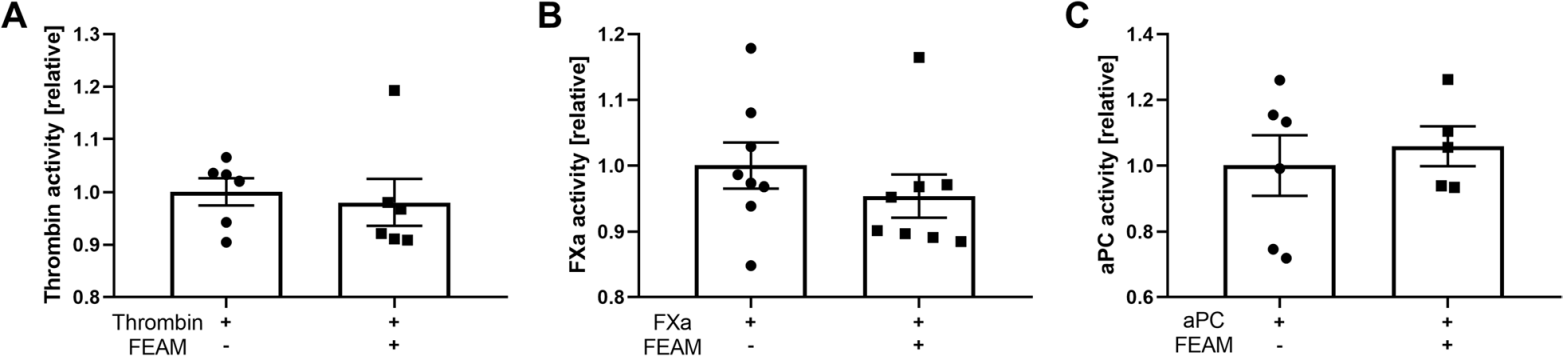
FEAM's direct effect on coagulation factors: FEAM has no significant effect on activities of commercial **A** Thrombin. *N* = 6. **B** FXa. *N* = 8. **C** aPC. *N* = 5–6. Results are presented as mean ± SEM

First, to evaluate the direct effect of FEAM on central coagulation factors, it was applied to commercial thrombin, FXa, and aPC, and their residual activities were measured. FEAM was found to have no significant effect on thrombin, FXa, and aPC activities (Fig. 3A–C).

**Fig. 4:**
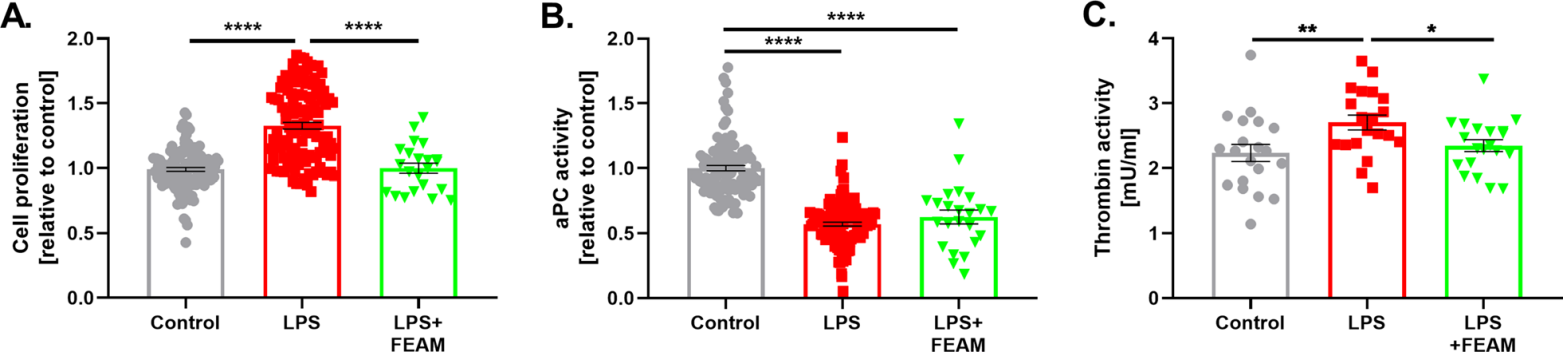
The effects of FEAM on LPS-induced neuroinflammation in N9 microglia cell-line: **A** Cell proliferation: LPS treatment caused significantly higher cell proliferation, compared to control. LPS model cells, that were treated with FEAM, showed significantly lower proliferation compared to untreated LPS cells. *N* = 22–120. **B** aPC activity: LPS treatment caused significantly lower aPC activity, compared to control. FEAM treatment had no significant effect on aPC activity in LPS model cells. *N* = 23–118. **C** Thrombin activity: LPS treatment caused significantly higher thrombin activity, compared to control. FEAM treatment significantly decreased thrombin activity compared to untreated LPS cells. *N* = 20. Results are presented as mean ± SEM, *p < 0.05, **p < 0.01, ****p < 0.0001

Next, we studied the FEAM-induced molecular changes in N9 cells in an LPS-induced inflammation model (Fig. 4). As expected, cells that were treated with LPS for 24 h showed significantly higher proliferation, compared to control (1.32 ± 0.02, 1.00 ± 0.01, respectively, *p* < 0.0001, Fig. 4A). Increased cell proliferation was decreased significantly, when FEAM treatment was applied simultaneously with LPS for 24 h, compared to LPS only (1.00 ± 0.04, 1.32 ± 0.02, respectively, *p* < 0.0001, Fig. 4A). In addition, LPS treatment applied for 24 h caused significantly lower aPC activity in N9 cells compared to untreated cells (0.57 ± 0.01, 1.00 ± 0.02, respectively, *p* < 0.0001, Fig. 4B). The FEAM molecule (that was applied simultaneously with LPS) did not prevent the effect of LPS treatment on aPC activity in these experimental settings (Fig. 4B). We also found that in contrast to aPC activity, thrombin activity increased significantly following LPS treatment compared to control and decreased significantly in combination with FEAM (2.23 ± 0.13, 2.7 ± 0.11, 2.35 ± 0.09 mU/ml for control, LPS and LPS + FEAM treatment, *p* = 0.01 by one-way ANOVA and *p* = 0.02 by t-test, respectively, Fig. 4C).

**Fig. 5:**
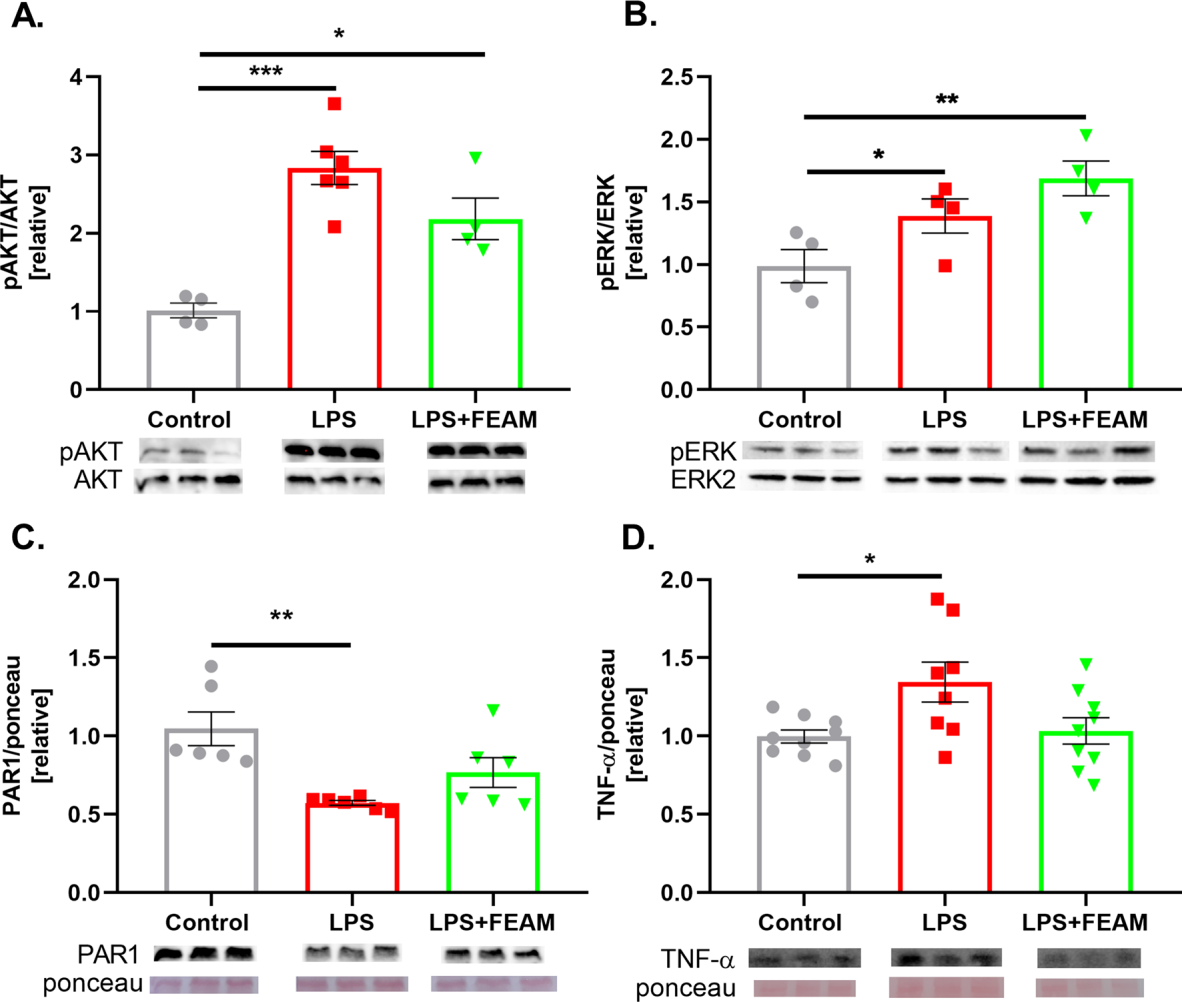
The effects of FEAM on LPS-induced neuroinflammation in N9 microglia cell-line: **A** protein kinase B (pAKT) protein levels significantly increased following LPS treatment. This effect was partially prevented by FEAM. *N* = 4–6. **B** phosphorylated extracellular regulated kinase (pERK) protein levels significantly increased following LPS treatment and further increased following FEAM treatment. *N* = 4. **C** PAR1 protein levels were significantly reduced following LPS treatment and were partially recovered by FEAM. *N* = 6. **D** Tumor-necrosis factor α (TNF-α) protein levels significantly increased following LPS treatment. This increase was prevented by FEAM. *N* = 8–9. Results are presented as mean ± SEM, *p < 0.05, **p < 0.005, ****p < 0.0002

We continued with the protein analysis of major coagulation and inflammation factors. To examine PAR1 and EPCR activation, we measured phosphorylated protein kinase B (pAKT) and phosphorylated extracellular regulated kinase (pERK) protein levels. We found elevated levels of pAKT compared to control following LPS treatment. This elevation was partially prevented by FEAM (1.00 ± 0.09, 2.83 ± 0.81, 2.18 ± 0.26, for control, LPS, and LPS + FEAM treatment, *p* < 0.0002 and *p* < 0.05, respectively, Fig. 5A). Similar to pAKT, pERK levels increased significantly following LPS treatment, but in contrast to pAKT, a further increase in pERK was measured with FEAM treatment (0.99 ± 0.13, 1.39 ± 0.14, 1.69 ± 0.14, for control, LPS and LPS + FEAM treatment, *p* < 0.04 by t-test and *p* < 0.01 by one-way ANOVA, respectively, Fig. 5B). In addition, the significant decrease in PAR1 levels following LPS treatment (0.75 ± 0.01, 1.04 ± 0.11, for LPS and control, respectively, *p* = 0.003, Fig. 5C) was prevented by FEAM to a certain degree (0.77 ± 0.09, *p* = 0.07, Fig. 5C). The significant elevation in the pro-inflammatory factor tumor necrosis factor-alpha (TNF-α) following LPS treatment (1.35 ± 0.12, 1.00 ± 0.04 for LPS and control, respectively, *p* = 0.02, Fig. 5D) was prevented by FEAM as well (1.03 ± 0.06, *p* = 0.94, Fig. 5D).

#### FEAM treatment in mTBI in-vivo model for neuroinflammation

**Fig. 6:**
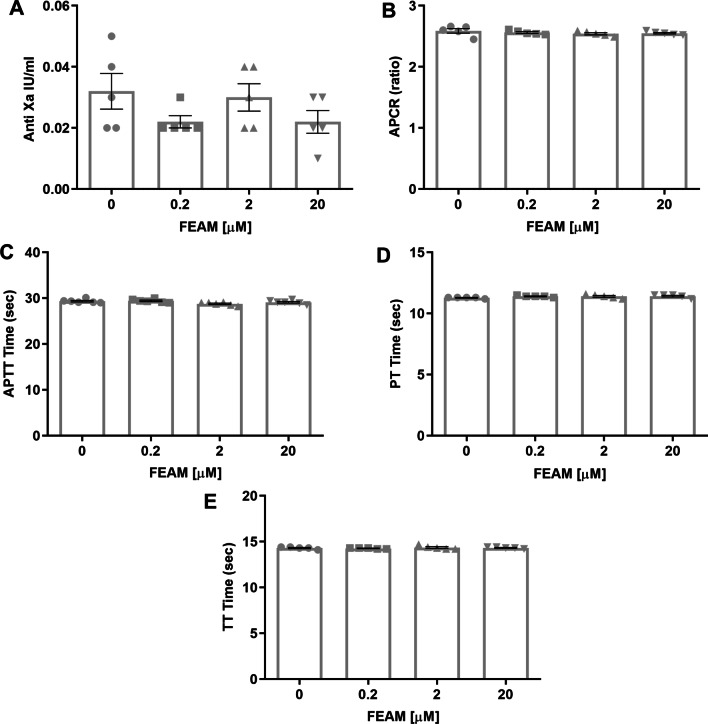
Safety coagulation tests for FEAM treatment: FEAM was found to not affect coagulation profile, including anti- Xa levels (**A**), activated protein C resistance (APCR) (**B**), activated partial thromboplastin time (APTT) (**C**), prothrombin time (PT) (**D**), and thrombin time (TT) (**E**). *N* = 5–6. Results are presented as mean ± SEM

Although FEAM was found to have no significant effect on the activities of commercial thrombin, FXa, and aPC, its design rationale implies modification of the aPC/EPCR pathway. Thus, it was important to study its effect on coagulation function, to better define the therapeutic window. We conducted an anti-Xa assay, APCR assay, PT, aPTT, and TT standard tests, in concentrations approximately ten-fold above and ten-fold below the in-vitro work concentration, and found no clinically significant effects for FEAM at tested concentrations in all studies assessed (Fig. 6). This strongly supports FEAM to be safe in terms of bleeding and blood coagulation disruption.

Next, we examined the effect of FEAM treatment in mTBI mice as an in-vivo model for neuroinflammation. We examined hippocampal molecular modifications as well as body weight, thrombin activity and behavioral changes at four points in time, and sensory changes at the latest point in time.

**Fig. 7:**
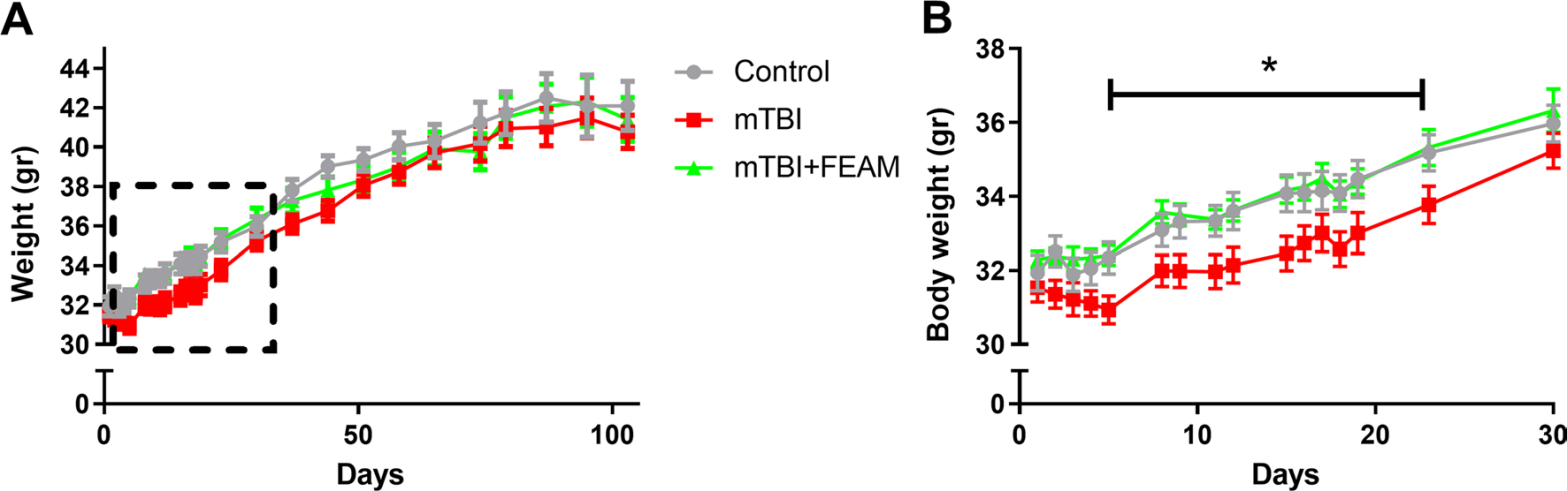
FEAM treatment in mTBI model: **A** The average weight of mice during the experiment. **B** The average weight of mice – day1 to 30: mTBI mice showed a significant decrease in body weight, between days 5 to 23, compared to the control and mTBI + FEAM group. *N* = 14. Results are presented as mean ± SEM, *p < 0.05

As can be seen in Fig. 7A and B, FEAM treatment prevented the bodyweight drop that was detected in mTBI mice between days 5 to 23, post mTBI (*p*-mTBI) (F(2,39) = 3.628, *p* = 0.03 for treatment).

**Fig. 8:**
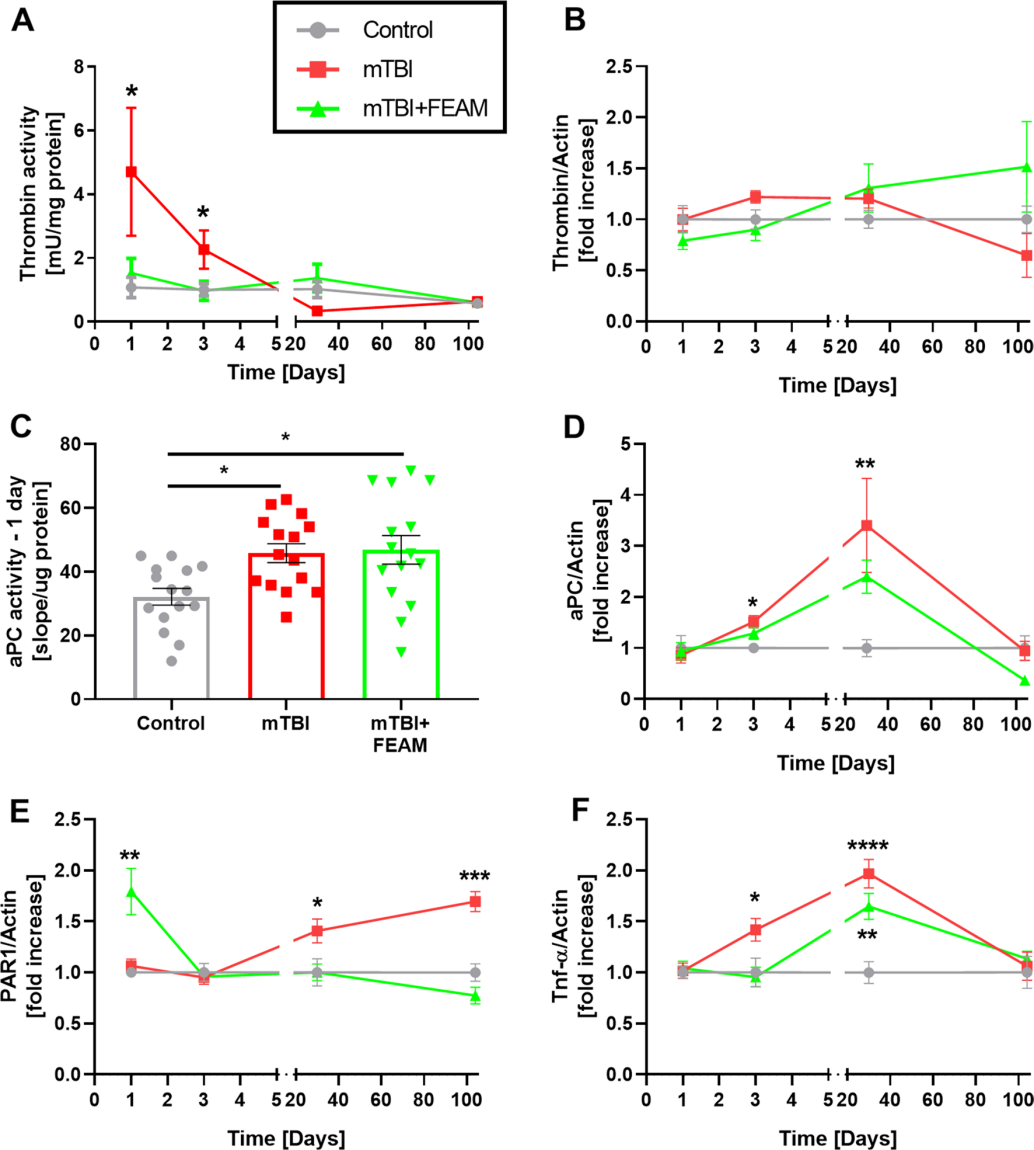
Activity and proteins present in the hippocampus of mTBI model: **A** Higher thrombin activity was found in the brain of mTBI group mice on days 1 and 3. No differences between the groups were found on days 30 and 104. *N* = 8–15. **B** No higher thrombin protein levels were found in mTBI mice brain p-mTBI. *N* = 3–11. **C** Higher aPC activity levels were found in mTBI and mTBI + FEAM mice brain 1-day p-mTBI. *N* = 15. **D** Higher aPC protein levels were found in mTBI mice brain 3- and 30-days p-mTBI. *N* = 3–11. **D** Higher PAR1 protein levels were found in mTBI + FEAM mice brain 1-day p-mTBI. Higher PAR1 protein levels were found in mTBI mice brain 30- and 104-days p-mTBI. *N* = 3–11. **E** Higher TNF-α protein levels were found in mTBI mice brain 3 days p-mTBI. Higher TNF-α protein levels were found in mTBI and mTBI + FEAM mice brain 30 days p-mTBI. *N* = 2–11. Results are presented as mean ± SEM, *p < 0.05, **p < 0.01, ****p < 0.0001

At the early time points (1 and 3 days), significantly higher thrombin activity level was detected in the hippocampus of mTBI group mice compared to control (Day 1: 4.70 ± 2.00, 1.08 ± 0.32 mU/mg protein, respectively, *p* = 0.04. Day 3: 2.26 ± 0.60, 1.00 ± 0.23 mU/mg protein, respectively, *p* = 0.05 Fig. 8A). At both time points this activity was found to be similar to control following FEAM treatment (Day 1: 1.53 ± 0.46 mU/mg protein, *p* = 0.9, Day 3: 0.97 ± 0.30 mU/mg protein, *p* = 0.9, for mTBI treated with FEAM, Fig. 8A). Despite increased thrombin activity 24 and 72 h following mTBI, no significant increase in thrombin protein levels was found *p*-mTBI compared to control (Fig. 8B), indicating changes in prothrombin activation and not in thrombin expression. The most significant changes in thrombin activity were measured 1-day p-mTBI (Fig. 8A), therefore we examined the changes in aPC activity at this time point. As can be seen in Fig. 8C, aPC activity increased significantly in mTBI and mTBI + FEAM groups compared to control (45.81 ± 2.6, 46.85 ± 4.5, and 32.13 ± 2.6, respectively, *p* < 0.015, Fig. 8C). Interestingly, like thrombin, no significant increase in aPC protein levels was found at this time point (Fig. 8D). At the later points, significantly increased aPC protein levels were found on days 3 and 30 p-mTBI compared to control (Day 3: 1.52 ± 0.12, 1.00 ± 0.08-fold increase, respectively, *p* = 0.02, Day 3: 3.4 ± 0.92, 1.00 ± 0.17-fold increase, respectively, *p* = 0.004 Fig. 8D). In addition, PAR1 protein levels were significantly increased in the mTBI + FEAM group one day following trauma compared to control (1.79 ± 0.23, 1.00 ± 0.05 fold-increased, respectively, *p* = 0.003, Fig. 8E) and in mTBI group 30 and 104 days *p*-mTBI compared to control (Day 30: 1.41 ± 0.11, 1.00 ± 0.13 fold-increased, respectively, *p* = 0.05, Day 104: 1.7 ± 0.1, 1.00 ± 0.09 fold-increased, respectively, *p* = 0.001, Fig. 8E). The pro-inflammatory cytokine TNF-α was significantly increased in the mTBI group compared to control on days 3 and 30 p-mTBI (Day 3: 1.42 ± 0.11, 1.00 ± 0.14 fold-increase, respectively, *p* = 0.05, Day 30: 1.97 ± 0.14, 1.00 ± 0.12, *p* < 0.0001, Fig. 8F), and in the mTBI + FEAM group only at 30 days p-mTBI (1.65 ± 0.13, p = 0.002, Fig. 8F).

**Fig. 9:**
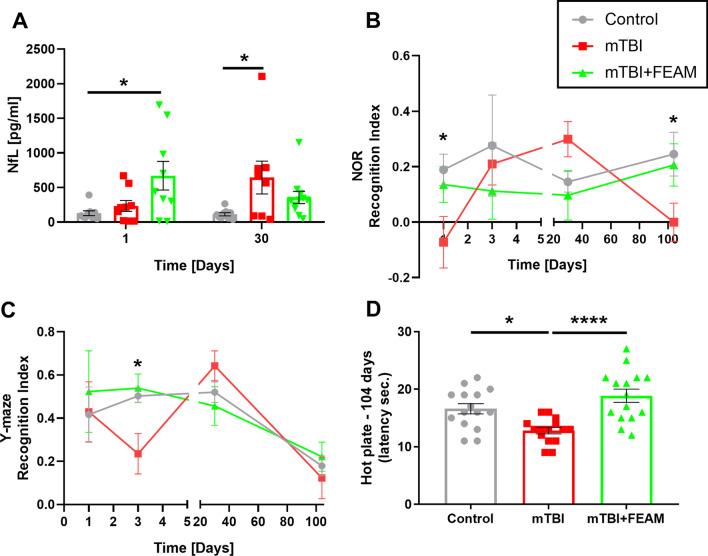
NfL serum levels, behavioral tests, and sensory evaluation: **A** mTBI + FEAM mice showed significantly higher NfL serum levels 1-day p-mTBI. mTBI mice showed significantly higher NfL serum levels 30 days p-mTBI. *N* = 8–11. **B** mTBI mice showed significantly lower recognition index on NOR test at 1 day and 104 days p-mTBI. *N* = 4–15. **C** mTBI mice showed significantly lower recognition index on Y-maze 3 days p-mTBI. *N* = 7–15. **D** mTBI mice showed significantly higher heat sensitivity (lower latency to response) 104 days p-mTBI, compared to the control and mTBI + FEAM group. *N* = 15. Results are presented as mean ± SEM, *p < 0.05, ****p < 0.0001

To evaluate neuronal damage p-mTBI we examined NfL in mice serum at early and late time points (1 and 30 days, respectively). Significant higher NfL serum levels were found at the early-stage p-mTBI in the mTBI + FEAM group, compared to control (670.5 ± 206.8, 129.5 ± 34.8 pg/ml, respectively, *p* = 0.017, Fig. 9A), but not in the mTBI only group (233 ± 78.7, 129.5 ± 34.8 pg/ml, respectively, *p* = 0.93, Fig. 9A). At the late stage (30 days), mTBI mice (but not mTBI + FEAM mice), had significantly higher NfL levels in the serum, compared to control (644.2 ± 237.9, 137.4 ± 22.9 pg/ml, respectively, *p* = 0.024, Fig. 9A). Neuronal damage can cause impaired memory and therefore we conducted two behavioral tests to evaluate different types of memory: NOR for recognition memory and Y-maze for spatial memory. As can be seen in Fig. 9B, recognition memory (as indicated by a lower recognition index) was significantly impaired by the head trauma both at the early and the late time points (Day 1: 0.19 ± 0.06, − 0.07 ± 0.09, for control and mTBI, respectively, *p* = 0.03, Day 104: 0.25 ± 0.08, − 0.0002 ± 0.07 for control and mTBI, respectively, *p* = 0.05, Fig. 9B). In contrast, spatial memory was significantly impaired only at the early time point as indicated by the lower recognition index found in the mTBI group 3 days *p*-mTBI (0.50 ± 0.03, 0.23 ± 0.09 for control and mTBI, respectively, *p* = 0.02, Fig. 9C). Evaluation of hyper/hypoalgesia was conducted by a hot plate at day 104. mTBI mice were significantly more sensitive to heat compared to control and to the mTBI + FEAM group (12.8 ± 0,56, 16.6 ± 0.89, 18.87 ± 1.14 s for mTBI, control, and mTBI + FEAM, respectively, *p* = 0.01, *p* < 0.0001, Fig. 9D).

## Discussion

In recent years there has been a major effort to develop compounds that increase aPC activity, to treat neuroinflammation [33, 34]. In the present study, we used novel molecules, which were specifically designed to modify the FVIIa-aPC-EPCR pathway, to achieve an elevation in aPC activity, a lowering of thrombin activity, and a reduction in microglial proliferation. A microglia cell-based assay was used to select a lead molecule, which was then found to have beneficial effects in both an in-vitro cell-based LPS model and a whole animal mTBI neuroinflammatory model.

Microglia proliferate under pathological conditions, including Alzheimer’s disease [35] and stress [36], and can potentially harm brain tissue [37]. aPC differentially affects cell proliferation. It induces proliferation in endothelial cells via the mitogen-activated protein kinase pathway and PAR1 [38]. aPC also inhibits proliferation of T-cells during inflammation in an LPS model [39]. aPC has been found to suppress microglia activation in an animal model of multiple sclerosis, known as a prototype of brain inflammatory diseases [40]. Among the molecules we presently developed and studied, FEAM2 was found to reduce the proliferation index of the microglial cells and indeed to elevate aPC activity. Since FEAM molecules were designed specifically to modulate the binding of aPC and FVII to their major receptor, EPCR, we suggest these effects involve specific mechanisms that modulate this receptor. Several subsequent findings in the present study indicate a modulatory role for FEAM on EPCR including effects on cell signaling molecules such as AKT, the lack of a direct effect on coagulation factors, and the relatively long-term effect on the whole brain. The ability of most FEAM compounds to elevate aPC strengthens the hypothesis that these compounds bind to EPCR and lead to a feedback loop by which the microglia sense decreased aPC and increase the cellular production of PC which is subsequently activated. Based on this effect FEAM2 was presently chosen as a leading molecule for further evaluation and was designated “FEAM”. This approach was further strengthened by the clear effect of FEAM in counteracting the proliferative effects of LPS on a microglial cell culture model. Evaluation of FEAM effects on purified coagulation factors indicates that FEAM has no direct effect on thrombin, FXa, and aPC activity, and thus the increased aPC and reduced thrombin activity induced by FEAM do not involve a direct effect on coagulation factors but rather a modulatory effect on EPCR. These data further support that FEAM’s mechanism of action is cellular-based and receptor-mediated through EPCR/PAR1 and may potentially be safe for clinical neuroinflammation studies. Thus, the development of FEAM and similar molecules offers the unique potential of lowering thrombin and increasing aPC activities without anti-coagulation associated side effects.

In the in-vitro LPS model, the effect of FEAM was mainly to prevent the LPS-induced cell proliferation and the increased thrombin activity. FEAM did not affect the LPS-induced decreased aPC activity. PAR1, the central cellular thrombin receptor, undergoes a conformational change following activation by thrombin or by the aPC/EPCR complex [41]. This promotes interactions with various G proteins that initiate intracellular signaling pathways, including AKT and ERK [24, 42]. In addition, activated PAR1 undergoes internalization which contributes to signal termination [43]. Our results demonstrate LPS induced elevated pERK and pAKT levels, plausibly caused by higher thrombin activity. LPS induced decreased PAR1 protein levels that indicate internalization of PAR1. LPS-induced increase in TNF-α was prevented by FEAM. Induction of TNF-α production by thrombin was previously reported [44]. Thus, this specific FEAM effect may be attributed to thrombin inhibition. Thrombin inhibition may also explain the normalization of proliferation, decreased PAR1 protein levels, increased pAKT, and TNF-α protein levels. In the in-vivo study, we found that mTBI causes a significant increase in thrombin activity 1 and 3 days p-mTBI, consistent with previous studies [15]. As in the in-vitro model, this increased activity was blocked by FEAM. Interestingly, thrombin protein levels did not change significantly p-mTBI. This discrepancy may have several explanations: First, the fluorescence method for thrombin activity measurement has a higher sensitivity compared to western blot analysis. Second, western blot detects mostly the membrane-bound thrombin while the fluorescence method detects the soluble thrombin as well. Finally, elevated thrombin activity may represent increased affinity of the thrombin active site, which cleaves PAR1 and the corresponding sequence of the fluorescence substrate and not due to increased protein level.

FEAM prevents weight loss p-mTBI between days 5 to 23. Weight loss following inflammation has been observed previously [45] and attributed, among other factors, to elevated TNF-α levels by several suggested mechanisms [46] and it is suggested that its protective effect on weight may be mediated by TNF-α inhibition. Coagulation and inflammation are closely linked, and thrombin is known to have several pro-inflammatory properties. Therefore, it is not surprising that the inflammatory cytokine TNF-α protein levels are found to be increased just after the increase of thrombin p-mTBI. Similar to effects on aPC protein levels, FEAM only partially prevented the increase in TNF-α.

In the mTBI mouse model, we found increased aPC activity 1-day p-mTBI, which is consistent with our previous studies [26]. The increased aPC activity was found at the time point at which the most significant increase in thrombin activity was measured. We may hypothesize that this elevation in aPC represents an intrinsic attempt of the brain tissue to counteract the neuroinflammatory effects of the trauma. FEAM counteracts the detrimental increase of thrombin activity without lowering aPC levels at this early stage following trauma. Interestingly, aPC levels in the microglia cell model decreased in response to LPS and increased in the mTBI model. The brain contains a variety of cells, each of them contributing to the aPC balance. aPC activity may differentially change in various cell types. Thus the decreased aPC activity found in microglia cell culture in response to LPS may be difficult to assess in a specimen containing all brain cell types. Thrombin together with thrombomodulin activates PC to aPC as a negative feedback loop. This may explain our results indicating a time lag between the high levels of thrombin activity, that were detected at days 1 to 3 p-mTBI and aPC protein levels that were detected at 3 and 30 days following trauma and again supports a long-term modulatory effect of FEAM through EPCR. Thus, the finding that FEAM significantly prevents the increased thrombin activity, but only partially prevents the increased aPC levels and activity, may indicate a promising therapeutic strategy.

In the mTBI model, we did not find any changes in PAR1 at the early stages (1 and at 3 days p-mTBI), but we did find significantly increased PAR1 levels at the later stages (day 30 and 104 days p-mTBI). Interestingly, FEAM treatment caused a significant increase in PAR1 levels 1-day p-mTBI and normalized its levels through the rest of the experiment. The early increased PAR1 levels found in the hippocampus of the FEAM-treated mice may represent a protective mechanism. This mechanism may be induced in response to the very-early damage associated with PAR1 decrease known to occur right after head trauma (5 min and 1 h followed the trauma)[17]. The late phase increased PAR1 levels found in the mTBI mice, but not in the FEAM-treated mice, may be attributed to long-term processes such as gliosis.

To evaluate neuronal damage, we measured NfL levels in the serum of model mice at early and late time points (1 and 30 days p-mTBI). NfL is a biomarker in several neurological disorders [47] and its levels are found to be increased in patients with suspected mTBI with neuroimaging findings [48]. mTBI mice had increased NfL levels 30 days p-mTBI indicating ongoing neuronal damage at this late time point which was partially blocked by FEAM. In contrast, FEAM-treated mice showed significantly higher NfL levels 1-day p-mTBI compared to control. We suggest that the long-term effects of FEAM represent a true neuroprotective anti-inflammatory mechanism while the early effect may represent, possibly beneficial, early clearance of neuronal debris.

As was found before, increased thrombin following mTBI is related to cognitive deficits [17]. In addition, it was previously reported that neuroinflammation can lead to chronic pain, through proinflammatory cytokines and chemokines production [49]. Indeed, the current data shows that mTBI caused impaired recognition memory at 1 and 104 days following the trauma. Also, impaired spatial memory was found 3 days p-mTBI. This data suggests that besides the immediate damage (as seen on days 1 and 3 p-mTBI), increased thrombin activity and inflammation also contribute to the long-term damage that was detected 104 days following mTBI. This long-term damage is further supported by the increased NfL levels measured 30 days p-mTBI. Furthermore, increased heat sensitivity was found 104 days p-mTBI. FEAM completely restored the impaired recognition and spatial memory, and heat sensitivity, and this is at least partially explained by thrombin activity normalization and reduction of the inflammation. Previous studies show that TNF-α deficient mice present better memory function in the short term, but worse memory function in the long term, which indicates that TNF-α is harmful at the first stages of the trauma but necessary in the process of recovery [50]. In the present study, FEAM blocked the increase in TNF-α levels at 1 and 3 days following the trauma, but the levels remain high 30 days following the trauma. This suggests that the current protocol of FEAM treatment, which was administrated at the early points in time (days 0–21), affects the early TNF-α deleterious behavioral effect, but has no influence on its later protective effects.

### Future study

Further studies in specific cell types, such as astrocytes and neurons and neuroinflammatory models is needed to evaluate the effect of FEAM and define its mechanism of action. Better understanding of the receptor-mediated mechanism behind the modifications of thrombin and aPC activity will improve the therapeutic potential of FEAM. Indeed, the development of more stable derivatives with better delivery and safety characteristics will potentially advance this aim of therapeutic targets for intervention in neuroinflammation.

## Conclusions

To conclude, in this study we found that neuroinflammation leads to increased thrombin activity and decreased aPC activity. As a result, PAR1 is over-activated and TNF-α is increased seemingly through the pERK/pAKT pathway. Our novel molecule FEAM was able to block most of the negative effects of neuroinflammation in-vitro and in-vivo, presumably through EPCR-PAR1 interactions.

## Data Availability

The datasets used and/or analysed during the current study are available from the corresponding author on reasonable request.

## Abbreviations

FEAM: Factor VII, EPCR, aPC Modulators
PAR1: Protease-activated receptor 1
aPC: Activated protein C
EPCR: Endothelial protein C receptor
GLA: Gamma carboxylated
pERK: Phosphorylated extracellular regulated kinase
pAKT: Protein kinase B
TNF-α: Tumor necrosis factor alpha
NOR: Novel object recognition
mTBI: Minimal traumatic brain injury
DMEM: Dulbecco’s modified Eagle’s medium
LPS: Lipopolysaccharide
NAPAP: Alpha-naphthylsulphonylglycyl-4-amidinophenylalanine piperidine
PT: Prothrombin time
aPTT: Activated partial thromboplastin time
TT: Thrombin time
anti-Xa: Anti-factor Xa
I.P.: Peritoneally
PBS: Phosphate-buffered saline
RIPA: Radioimmunoprecipitation assay
BCA: Bicinchoninic acid
NfL: Neurofilament
p-mTBI: Post mTBI
TF: Tissue factor
